# Higher-order functional brain networks and anterior cingulate glutamate + glutamine (Glx) in antipsychotic-naïve first episode psychosis patients

**DOI:** 10.1038/s41398-024-02854-7

**Published:** 2024-04-10

**Authors:** Jose O. Maximo, Frederic Briend, William P. Armstrong, Nina V. Kraguljac, Adrienne C. Lahti

**Affiliations:** 1 Department of Psychiatry and Behavioral Neurobiology, University of Alabama at Birmingham, Birmingham, AL USA; 2 UMR1253, iBrain, Université de Tours, Inserm, Tours, France

**Keywords:** Schizophrenia, Diagnostic markers

## Abstract

Human connectome studies have provided abundant data consistent with the hypothesis that functional dysconnectivity is predominant in psychosis spectrum disorders. Converging lines of evidence also suggest an interaction between dorsal anterior cingulate cortex (dACC) cortical glutamate with higher-order functional brain networks (FC) such as the default mode (DMN), dorsal attention (DAN), and executive control networks (ECN) in healthy controls (HC) and this mechanism may be impaired in psychosis. Data from 70 antipsychotic-medication naïve first-episode psychosis (FEP) and 52 HC were analyzed. 3T Proton magnetic resonance spectroscopy (1H-MRS) data were acquired from a voxel in the dACC and assessed correlations (positive FC) and anticorrelations (negative FC) of the DMN, DAN, and ECN. We then performed regressions to assess associations between glutamate + glutamine (Glx) with positive and negative FC of these same networks and compared them between groups. We found alterations in positive and negative FC in all networks (HC > FEP). A relationship between dACC Glx and positive and negative FC was found in both groups, but when comparing these relationships between groups, we found contrasting associations between these variables in FEP patients compared to HC. We demonstrated that both positive and negative FC in three higher-order resting state networks are already altered in antipsychotic-naïve FEP, underscoring the importance of also considering anticorrelations for optimal characterization of large-scale functional brain networks as these represent biological processes as well. Our data also adds to the growing body of evidence supporting the role of dACC cortical Glx as a mechanism underlying alterations in functional brain network connectivity. Overall, the implications for these findings are imperative as this particular mechanism may differ in untreated or chronic psychotic patients; therefore, understanding this mechanism prior to treatment could better inform clinicians.

**Clinical trial registration**: Trajectories of Treatment Response as Window into the Heterogeneity of Psychosis: A Longitudinal Multimodal Imaging Study, NCT03442101. Glutamate, Brain Connectivity and Duration of Untreated Psychosis (DUP), NCT02034253.

## Introduction

Studies of the human connectome have provided an abundance of data consistent with the hypothesis that dysconnectivity, the aberrant integration of functional brain networks, is fundamental in psychosis spectrum disorders [1–3]. The dysconnectivity model posits that psychosis can be better understood from a neurobiological perspective since N-methyl-d-aspartate receptor (NMDAR) functioning moderates disturbances in the synaptic efficacy affecting the excitation/inhibition balance thus affecting the intrinsic (local) and extrinsic (long-range) connectivity of functional brain networks [4]. Consistent with this, recent studies have found that cortical glutamate plays an important role in modulating the blood-oxygen-level-dependent (BOLD) signal [5] and functional connectivity [6], and pharmacological challenge studies have also found a disruption in functional brain networks following experimentally induced NMDAR hypofunction in healthy human subjects [6–8]. Taken together, it is plausible that cortical glutamate may play an important modulatory role in functional brain network dysconnectivity observed in psychosis spectrum disorders [9–12].

We recently reported the results of a multimodal neuroimaging study in antipsychotic-medication naïve first episode psychosis patients (FEP) where we used proton magnetic resonance spectroscopy (1H-MRS) to quantify cortical glutamate + glutamine (Glx) in the dorsal anterior cingulate cortex (dACC) and resting-state functional magnetic resonance imaging (fMRI) to assess functional connectivity in the salience network (SN, which spans across the dACC and the anterior insula) [13]. Our principal finding, reduced salience network connectivity in FEP and the lack of a robust relationship between glutamate and connectivity that is present in healthy subjects is in agreement with the dysconnectivity hypothesis from our findings of dACC cortical Glx playing a modulatory role in functional brain connectivity in psychosis at a local level.

Because a number of functional networks that support higher-order cognitive functioning have been implicated in the schizophrenia pathophysiology [14–19], and cortical Glx may not only have local effects as previously shown by our previous study but may also modulate long-range functional connectivity of higher-order functional brain networks in FEP as this has been previously observed in healthy individuals [20, 21], we chose to expand this line of investigation using an overlapping sample and interrogate putative associations between dACC cortical Glx and relevant higher-order functional brain networks: default mode (DMN), executive control (ECN), and dorsal attention network (DAN). These higher-order functional brain networks were chosen as they are responsible for specific cognitive functioning known to be impaired in schizophrenia [22]. We investigated both correlations and anti-correlations of these higher-order functional brain networks. Anticorrelations were considered in our analyses because the presence of anticorrelated networks has been described at rest [23] and functional connectome data-based modeling has highlighted the importance of anticorrelations for optimal characterization of higher-order functional brain network connectivity [24]. Additionally, reduced anticorrelations may be regarded as signs of decreased brain flexibility/inhibition to react upon external stimuli consequently altering cortical glutamate activity [25, 26]. We hypothesized that dysconnectivity in the DMN, ECN, and the DAN is present for both correlations and anticorrelations in antipsychotic-naïve FEP and that there would be a strong relationship between dACC cortical Glx and functional connectivity in higher-order functional brain networks in healthy controls (HC), but this relationship would be weaker in FEP.

## Materials and methods

### Participants

A total of 127 (HC = 54; FEP = 73) subjects participated in this study. FEP patients were recruited from outpatient clinics, inpatient units, and the emergency room at the University of Alabama at Birmingham (UAB). Studies were approved by the UAB Institutional Review Board, and written informed consent was obtained before enrollment (patients had to be deemed competent to provide consent) [27]. Exclusion criteria were major neurological or medical conditions, history of significant head trauma, substance use disorders (excluding nicotine and cannabis) within 1 month of imaging, more than five days of lifetime antipsychotic exposure, pregnancy or breastfeeding, and MRI contraindications. Consensus diagnoses were made according to DSM-5 criteria by two board-certified psychiatrists from all historical and direct assessment information available (ACL and NVK). Because FEP subjects were followed over several months, consensus diagnosis was also based on this longitudinal assessment. The Brief Psychiatric Rating Scale (BPRS) and Repeatable Battery for the Assessment of Neuropsychological Status (RBANS) were used to assess symptom severity and cognition [28, 29]. We also recruited healthy controls (HC) from flyers and ads within UAB and the emergency room where we recruited our patients. HCs were matched on age, gender, and parental socioeconomic status (SES). In addition to the above-outlined criteria, HCs with a personal history or a family history of a psychiatric illness in a first-degree relative were also excluded.

### Data acquisition

All imaging was performed on a 3T whole-body Siemens MAGNETOM Prisma MRI scanner equipped with a 20-channel head coil. A high-resolution T1-weighted structural scan was acquired for anatomical reference (MPRAGE: TR = 2400 ms; TE = 2.22 ms; inversion time = 1000 ms; flip angle = 8°; GRAPPA factor = 2; voxel size = 0.8 mm^3^). 1H-MRS data were collected from a voxel in the anterior cingulate cortex (27 × 20 × 10 mm^3^). Following automatic and manual shimming to optimize field homogeneity across the voxel, chemical shift selective (CHESS) pulses were used to suppress the water signal. Then, spectra were obtained using a point resolved Spectroscopy sequence (PRESS; TR/TE = 2000/80 ms, flip angle = 90°, vector size 1024, 96 averages [30, 31]). Moreover, 8 averages of unsuppressed water scans with the same acquisition parameters were acquired as an internal reference.

Finally, resting-state fMRI data was acquired in opposing phase encoding directions (anterior > posterior and posterior > anterior; TR = 1550 ms; TE = 37.80 ms; flip angle = 71°, FOV = 104 mm^2^; multi-band acceleration factor = 4; voxel size = 2 mm^3^; 225 volumes, and 72 axial slices). Subjects were instructed to look at a fixation cross, keep their eyes open, and let their mind wander.

### Data preprocessing

#### Proton magnetic resonance spectroscopy data

All spectra were analyzed in jMRUI version 6.0 using the AMARES algorithm ([32]. The model consisted of peaks for N-Acetyl aspartate (NAA), choline (Cho), Creatine (Cr + Cr2), Glx was modeled as a triplet (large peak with 2 small outer wings) as previously described [33]. After removing the residual water peak using the Hankel–Lanczos singular values decomposition filter, the amplitude of the center Glx peak was estimated and Glx levels were calculated relative to the unsuppressed voxel water and expressed in institutional units (I.U.) [34]. Metabolite levels were corrected for partial volume effects (i.e. gray and white matter voxel content) according to Gasparovic and colleagues [35, 36]; the fraction of cerebrospinal fluid, gray and white matter was calculated by segmentation of the T1-weighted images in SPM8.

Exclusion criteria for Glx were the failure of the fitting algorithm, signal-to-noise ratio (SNR) < 3, full width at half maximum (FWHM) > 0.1 ppm [37], and Cramer–Rao lower bounds (CRLB) > 20%.

#### Resting-state fMRI data

After discarding the first 10 volumes of each scan allowing for signal equilibration, susceptibility artifacts were corrected using spin echo field maps in FSL’s top-up, and then the 2 corrected fMRI runs were combined resulting in a single 4D image of 430 total volumes [38]. Data were analyzed using the CONN toolbox version 20a https://web.conn-toolbox.org [39]. Functional images were slice-timing and motion-corrected using rigid-body realignment, co-registered to the structural image, normalized to Montreal Neurological Institute (MNI) space, bandpass filtered (0.008 < *f* < 0.08 Hz), and spatially smoothed with a 4-mm FWHM Gaussian kernel.

Framewise displacement (FD) and percentage of censored data were then calculated [40]. Motion outliers as detected by the artifact detection (ART) toolbox were censored (composite volume-to-volume motion > 0.5 mm and intensity >3 SDs), and the six motion parameters derived from rigid-body realignment and their derivatives, as well as the first 5 component time series derived from CSF and white matter using aCompCor and corresponding derivatives, were regressed out from the signal. No global signal regression was performed as this can impact functional connectivity analyses.

### Statistical analyses

**Fig. 1:**
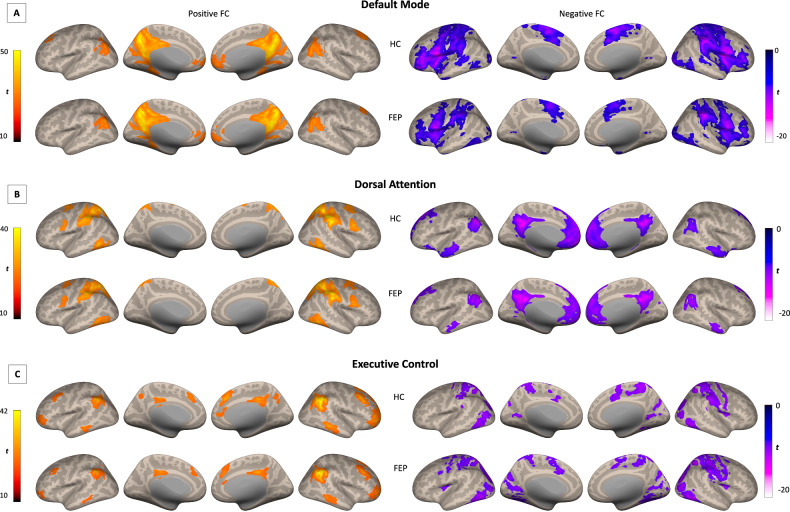
Within positive and negative FC maps for default mode, salience, and executive control networks for HC and FEP groups. Inflated brain renderings showing positive and negative FC maps for the **A** default mode, **B** dorsal attention, and **C** executive control network for each group. FC functional connectivity, HC healthy controls, FEP first episode psychosis.

For connectivity analyses, three regions of interest (ROIs) within the CONN toolbox were used: DMN (posterior cingulate cortex, PCC), DAN (right intraparietal sulcus, RIPS), and ECN (right posterior parietal cortex, RPPC). Residual time-series from each ROI were extracted and correlated with every other voxel in the brain, creating individual whole-brain *z*-transformed correlation maps (positive FC). Anticorrelation (negative FC) maps of each network were also computed for each network. To restrict all analyses within each functional brain network, functional network masks were created by thresholding average correlation maps for each group to a minimum value of *t* = 10 for positive FC and *t* = −20 for negative FC and a cluster size of 100 voxels. Then, average masks using all combined groups (HC + FEP) were created for each network (Fig. 1). Group analyses were then performed in CONN, but to restrict all analyses to their respective brain network masks, small volume correction (*p* < 0.01 defined by *α* = 0.05/4 networks of interest; also accounting for the salience network, for results see ref. [13]) were performed by running threshold-free cluster enhancement (TFCE) [41]. TFCE correction estimates a voxel-wise metric that captures the amount of cluster-like local spatial support for activation, combined with non-parametric permutation testing for inference. Age, sex, and FD were treated as covariates.

To examine how dACC Glx plays a role in connectivity in all three networks, Glx values were entered into regression analyses to predict connectivity on each network. This was done for both positive and anticorrelated areas. Average Glx-FC maps of each subject for each group were entered into group analyses to test for interaction effects (how Glx-FC associations differ between HC and FEP) while controlling for age, gender, and FD. Multiple comparison correction was performed as described above.

Associations between dACC Glx and clinical scores were assessed in our previous study and found no significant correlations. For this study, exploratory analyses were performed where FC data was extracted from clusters of significant group differences (clusters from Fig. 1) and correlated with BPRS and RBANS subscales scores while controlling for age, sex, and FD. Given that these analyses are exploratory in fashion, no correction for multiple comparisons was applied and results should be taken with caution.

## Results

### Demographics and clinical data

After excluding five subjects with noisy MRS data, seven subjects with missing data/scans (did not have resting state scans), and seven subjects with excessive head motion (<50% of data remaining after censoring or volume-to-volume motion > 0.5 mm), our sample included 122 (HC = 52; FEP = 70). Mean values for CRLB for each metabolite are also shown in Table 1.

**Table 1:** Demographics, clinical measures, and data quality.

	Groups (*N* = 122)		*P*-value
	HC (*n* = 52)	FEP (*n* = 70)	
*Demographic variables*			
Age (in years)	24.62 ± 6.28	24.03 ± 6.14	0.61
Sex (M/F)	34/18	44/26	0.77
^a^Parental occupation (SES)	4.46 ± 4.32	5.89 ± 4.71	0.33
*Race*			
White	32	21	–
African American	11	43	–
Asian	6	2	–
Hispanic	1	2	–
Pacific Islander	1	0	–
Other	1	2	–
^b^No. sf smokers (%)	8%	41%	
Smoking (Packs per day)	0.03 ± 0.09	0.24 ± 0.40	< 0.001
^c^No. of cannabis users (%)	0%	30%	
*Clinical variables*			
Diagnosis			
Schizophrenia	–	35	–
Schizoaffective disorder	–	11	–
Bipolar disorder with psychosis	–	3	–
Schizophreniform disorder	–	4	–
Psychosis NOS	–	13	–
Brief psychotic disorder	–	2	–
Major depressive disorder w/psychosis	-	2	–
Duration of untreated psychosis (in months)	–	21.43 ± 40.19	–
BPRS			
Positive (4-items)	–	11.42 ± 3.38	–
Negative	–	5.87 ± 3.21	–
Total	–	50.16 ± 11.62	–
^d^*RBANS*			
Immediate memory	100.21 ± 16.21	81.90 ± 17.83	<0.001
Visuospatial/Constructional	83.94 ± 13.51	75.00 ± 17.92	0.005
Language	98.15 ± 15.89	82.95 ± 16.87	< 0.001
Attention	102.53 ± 16.01	80.45 ± 17.01	< 0.001
Delayed memory	91.87 ± 8.86	77.32 ± 15.71	< 0.001
Total index	93.38 ± 11.56	74.15 ± 15.89	< 0.001
*Scan quality data*			
MRS			
Cramer–Rao lower bands (Glx)	3.63 ± 0.65	4.37 ± 1.58	0.002
Cramer–Rao lower bands (Cr)	0.80 ± 0.12	0.91 ± 0.19	0.001
Cramer–Rao lower bands (Cho)	1.16 ± 0.23	1.29 ± 0.32	0.014
Cramer–Rao lower bands (NAA)	0.72 ± 0.11	0.83 ± 0.17	< 0.001
GM fraction	72.18 ± 7.13	72.08 ± 5.56	0.93
WM fraction	12.86 ± 5.92	13.66 ± 6.04	0.47
CSF fraction	14.19 ± 3.95	14.26 ± 3.69	0.92
^e^*FC*			
Mean motion (in mm)	0.15 ± 0.06	0.19 ± 0.09	0.01
% of volumes after scrubbing	94.98 ± 5.59	91.10 ± 8.82	0.006

Mean ± standard deviation; data available for ^a^118 subjects. Ranks determined from Diagnostic Interview for Genetic Studies where a higher rank (lower numerical value) corresponds to higher socioeconomic status (SES); ^b^120 subjects; ^c^missing data for 8 patients; ^d^109 subjects; ^e^113 subjects; *P*-values are from *χ*^2^ and independent samples-*t* tests for differences between the groups.

*RBANS* repeatable battery for the assessment of neuropsychological status, *BPRS* brief psychiatric rating scale, *MRS* magnetic resonance spectroscopy, *GM* gray matter, *WM* white matter, *CSF* cerebrospinal fluid, *FC* functional connectivity.

Demographic data were compared between groups using a series of independent-sample *t*-tests and Chi-squares. HC and FEP were well matched in terms of age, gender, and parental socioeconomic status, but FEP patients smoked more often than HC (*p* < 0.001).

### Group differences in dACC Glx, network correlations and anticorrelations

As previously reported in ref. [13], dACC Glx did not differ between groups (*F*_1, 104_ = 0.57, *p* = 0.45).

**Fig. 2:**
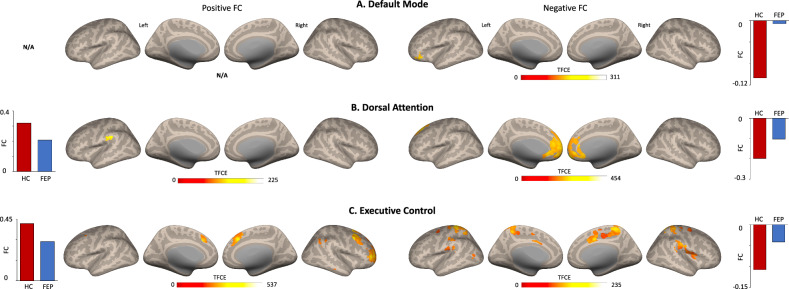
HC vs. FEP positive and negative FC for default mode, salience, and executive control networks. Clusters of significant group differences (positive and negative FC) for **A** default mode, **B** dorsal attention, and **C** executive control networks (all TFCE corrected). TFCE threshold-free cluster enhancement, FC functional connectivity, HC healthy controls, FEP first episode psychosis.

DMN-positive FC was largely similar between groups showing connectivity with traditional DMN regions such as bilateral angular gyrus, medial prefrontal cortex, and precuneus. Negative FC shows a different contrast with what is expected to resemble DAN and other areas. (Supplementary Table 1, Fig. 1A). Group differences revealed FEP showing reduced negative FC in the inferior frontal and orbital gyrus compared to HC (TFCE corrected, Table 2, Fig. 2A).

**Table 2:** Clusters of significant group differences in functional connectivity, separately for each network.

Network (seed)	Peak location, hemisphere	MNI coordinates			Cluster size	TFCE value
		*x*	*y*	*z*	(in voxels)	
*Default mode (PCC)*						
Positive FC	–	–	–	–	–	–
Negative FC	Inferior frontal gyrus, L	−44	26	−16	62	311.31
	Middle orbital gyrus, L	−34	42	−10	12	237.35
*Dorsal attention (RIPS)*						
Positive FC	Supramarginal gyrus, L	−60	−34	26	39	225.38
	Supramarginal gyrus, L	−60	−26	18	25	197.36
Negative FC	Mid orbital gyrus, R	2	56	−14	2130	454.42
Executive control (RPPC)						
Positive FC	Middle frontal gyrus, R	40	12	48	979	537.10
	Middle frontal gyrus, R	38	54	2	724	518.98
	Superior medial gyrus, R	4	26	40	401	504.75
	Angular gyrus, R	44	−56	52	58	255.91
	Inferior parietal lobule, R	50	−36	44	41	242.87
	Superior medial gyrus, R	8	36	58	19	209.39
	Middle frontal gyrus, L	−32	14	60	11	220.13
Negative FC	Superior parietal lobule, R	20	−52	64	612	235.79
	Postcentral gyrus, L	−26	−34	74	835	227.65
	Supplementary motor area, R	8	4	42	231	213.49
	Supramarginal gyrus, R	66	−20	20	449	183.77
	Supramarginal gyrus, L	−48	−36	26	122	146.92
	Precentral gyrus, R	26	−12	56	55	101.07
	Middle occipital gyrus, L	−36	−68	14	10	100.00
	Middle frontal gyrus, L	−24	−8	46	28	73.15
	Parahippocampal gyrus, L	−18	−2	−20	43	41.50
	Parahippocampal gyrus, R	18	0	−20	14	38.61
	Caudate nucleus, R	18	8	20	12	29.63

*PCC* posterior cingulate cortex, *RIPS* right intraparietal sulcus, *RPPC* right posterior parietal cortex, *R* right, *L* left, *MNI* Montreal Neurological Institute, *TFCE* threshold-free cluster enhancement, *FC* functional connectivity.

Similarly, groups showed DAN positive FC between intraparietal sulcus with bilateral middle temporal areas, frontal eye fields, and middle frontal areas and negative FC shows strong anticorrelations with DMN regions (Supplementary Table 1, Fig. 1B). Group differences revealed FEP showing reduced positive FC in left supramarginal gyrus and reduced negative FC in the medial orbital cortex compared to HC (TFCE corrected, Table 2, Fig. 2B).

Finally, ECN-positive FC spanned across the bilateral posterior parietal cortex, inferior middle temporal cortex, as well as dorsal and medial frontal cortices. As for negative FC, strong anticorrelations were found in sensory processing areas such as motor and visual regions (Supplementary Table 1 and Fig. 1C). In this network, positive FC was reduced in FEP in right parietal, temporal, and frontal regions while negative FC was stronger in the parietal, temporal and motor cortices when compared to HC (TFCE corrected, Table 2, Fig. 2C).

### Relationship between dACC cortical Glx and functional brain network connectivity

**Fig. 3:**
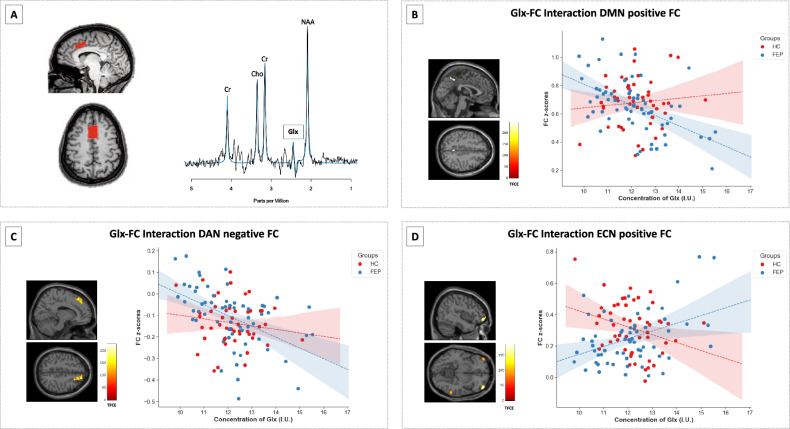
Sample voxel and spectra from ACC and Glx-FC interactions between HC and FEP. **A** Example voxel placement in the dorsal anterior cingulate cortex in one subject and its spectrum. The black line is a collected spectrum, and the blue line is a model fit. Clusters of group Glx-FC interactions in both HC and FEP in **B** default mode network (positive FC), **C** dorsal attention (negative FC), and **D** executive control network (positive FC; all TFCE corrected). BOLD signal was extracted for data scatterplot visualization purposes. Glx glutamate + glutamine, TFCE threshold-free cluster enhancement, FC functional connectivity, HC healthy controls, FEP first episode psychosis.

We found associations between dACC Glx and DMN positive FC in the medial prefrontal cortex, precuneus, and posterior cingulate cortex, and between Glx and DMN negative FC with the caudate nucleus in HC, while only two associations were found in the FEP group (Supplementary Table 2 and Supplementary Fig. 1). When directly comparing the relationships between dACC Glx and DMN positive FC between groups, we found a significant Glx-FC interaction within the bilateral precuneus. There were no significant Glx-FC interactions when examining Negative FC (TFCE corrected, Table 3, Fig. 3B).

**Table 3:** Clusters of significant group interactions (Glx-FC), separately for each network.

Network (seed)	Peak location, hemisphere	MNI Coordinates			Cluster size (in voxels)	TFCE value
		*x*	*y*	*z*		
Default mode (PCC)						
Positive FC	Precuneus, L	0	−48	46	34	245.50
Negative FC	–	–	–	–	–	–
Dorsal attention (RIPS)						
Positive FC	–	–	–	–	–	–
Negative FC	Superior frontal gyrus, R	16	32	52	420	225.39
Executive control (RPPC)						
Positive FC	Middle orbital gyrus, R	42	54	−6	117	184.32
	Inferior temporal gyrus, R	60	−46	−10	105	113.09
	Inferior frontal gyrus, L	−48	46	−12	115	111.56
Negative FC	–	–	–	–	–	–

*PCC* posterior cingulate cortex, *RIPS* right intraparietal sulcus, *RPPC* right posterior parietal cortex, *R* right, *L* left, *MNI* Montreal Neurological Institute, *TFCE* threshold-free cluster enhancement, *FC* functional connectivity.

In the DAN we found an association between dACC Glx and bilateral angular gyri and frontal areas in HC. We also found this correlation in FEP, but the spatial extent was more limited (Supplementary Table 2, Supplementary Fig. 2). An effect of Glx in DAN Negative FC was found in frontal pole areas and temporal gyri in both groups. However, when directly comparing the two groups no interaction effects were found between dACC Glx and positive FC, but when examining the relationship between dACC Glx and DAN negative FC, we found a a group interaction effect in the right superior frontal gyrus (TFCE corrected, Table 3, Fig. 3C).

Finally, we found an association between dACC Glx and ECN-positive FC in frontal and parietal areas in HC and FEP. ECN-negative FC showed a positive association with Glx in the postcentral gyrus and central opercular in HC and FEP as well (Supplementary Table 2 and Supplementary Fig. 3). When directly comparing the relationship between dACC Glx and ECN-positive FC between groups, we found three regions that showed an interaction effect in middle orbital, inferior frontal and temporal areas and no interaction effects were found between dACC Glx and negative FC (TFCE corrected, Table 3, Fig. 3D).

### Post-hoc brain-behavior analyses

Correlational findings between FC with all BPRS and RBANS subscales scores are shown in Supplementary Table 3. Furthermore, given the heterogeneity of FEP, we also explored these associations in schizophrenia patients only.

## Discussion

The main goals of our studies (current and [13]) were to characterize large-scale resting state networks, considering both correlations and anticorrelations, in antipsychotic-naïve patients suffering from a first psychotic episode and to investigate the role of dACC cortical Glx as a putative mechanism underlying alterations in functional connectivity. We first report that both groups show expected correlations (positive FC) and their corresponding anticorrelations (negative FC) on these brain networks from previous literature [42] with the exception of ECN where a large cluster in the visual cortex was found. Nonetheless, when comparing both groups, we found connectivity reductions in SN [13], DMN, ECN, and DAN in patients consistent with our hypothesis that dysconnectivity in large-scale functional networks supporting higher-order cognitive function is already evident in the early illness stages. We also found weaker negative FC of the DMN, ECN, and DAN in patients (but not in SN), extending the literature by demonstrating that spontaneous anticorrelated networks are already affected in antipsychotic naïve FEP. Importantly, Glx predicted positive and negative FC of all networks in healthy subjects and FEP. When directly contrasting the relationship between cortical Glx and connectivity between groups, we found inverse associations between these variables in patients compared to controls in both correlated and anticorrelated networks except in the DAN. Taken together, our data lend additional empirical support to the hypothesis that dACC cortical Glx plays a modulatory role in brain connectivity and point to a possible mechanism underlying dysconnectivity in psychosis not only at a local level as shown by our previous study, but at a global one.

Attempts have been made to characterize large-scale functional networks at rest in antipsychotic-naïve patients, despite evidence suggesting that functional network architecture is sensitive to neuromodulation [43–46] and dopamine targeting agents such as L-dopa, methylphenidate, and haloperidol affect functional connectivity in healthy subjects [43, 47, 48]. Our findings are generally consistent with network-level dysconnectivity evidence in FEP [49, 50] and demonstrate that alterations in patterns of correlations and anti-correlations in functional networks are not just a confound of antipsychotic medication exposure. Reduced connectivity in the DMN [49] and decreased resting state amplitudes of low-frequency fluctuations (ALFFs), a different measure of spontaneous synchronous neuronal activity, in areas of the DMN and ECN have also been reported in antipsychotic-naïve FEP [51]. A recent meta-analysis of higher-order resting state brain networks in early-stage psychosis patients reported widespread hypoconnectivity in the DMN, and mixed hypo- and hyperconnectivity in the ECN, but available data was much more limited for the ECN, and the authors concluded that further studies are required to draw any decisive conclusions about ECN connectivity in first episode psychosis [44]. In our study, only negative FC of the DMN showed a significant group difference in the same region reported by O’Neill and colleagues [44] while the ECN showed consistent reductions in connectivity and the greatest spatial extent of connectivity reductions across the three networks. Similar to Baker and colleagues who examined resting state ECN integrity across the psychosis spectrum, we did not see a relationship between aberrant connectivity and symptom severity [3]. Our findings of reduced positive and negative FC of the DAN in FEP compared to HC do not support our previous findings in unmedicated patients with schizophrenia where these showed increased resting-state brain connectivity [52]. Heterogeneity and illness chronicity in our previous sample could explain this discrepancy between findings (unmedicated vs. medication naïve FEP). In terms of our current findings, abnormal FC in the DAN may correspond to the concept of psychosis being a disorder of brain network organization, more specifically to these higher-order brain networks [44].

Anticorrelations are believed to be a product of healthy functional segregation, where excitatory and inhibitory mechanisms are at play. There is debate in the literature as to whether anticorrelations in resting state connectivity data are neurobiologically valid, or if they are artificially introduced by data preprocessing methods such as global signal regression (which was not performed in this study) [53]. Empirically testing the effects of different data processing strategies, Chai and colleagues concluded that anticorrelations observed in seed-defined resting-state networks cannot be fully attributed to artifacts introduced by global signal regression and might be neuronal in origin [54]. Lending additional support to interpreting anticorrelations as a manifestation of neuronal activity, simultaneous electrophysiological recordings of low field potentials in the cat homologs of task-positive and task-negative regions found that these were often anticorrelated [55] and computational simulations suggest the existence of spontaneous anticorrelated networks [56]. In schizophrenia, resting state anticorrelations have been found to be altered [57, 58] and shown moderate accuracy in differentiating patients from healthy controls using support vector machine approaches [59]. Interestingly, anticorrelations between the DMN and a task-positive network present in healthy subjects were absent in patients at high risk or ultrahigh risk for schizophrenia [60]. Attenuated anticorrelations between the DMN and ECN have also been observed during the performance of a working memory task in patients with schizophrenia [61], which is consistent with our finding of attenuated anticorrelations in functional brain networks. Taken together, data suggests a deficit of higher-order functional brain networks in psychosis spectrum disorders.

Because the administration of ketamine, a non-competitive NMDA receptor antagonist, can result in a comparable disruption of functional networks in HC, it is plausible that glutamate has a role in the altered organization of large-scale anticorrelated functional brain networks in schizophrenia [62]. Disruption in the excitation/inhibition balance may lead to an increase of excitability in cortical microcircuitry, affect connectivity in large-scale networks, and in turn result in behavioral abnormalities [63–65]. When empirically testing the role of dACC cortical Glx in the modulation of functional brain networks, we found that dACC Glx was associated with the strength of positive and negative FC in all networks in both groups. These associations have not only been confirmed in humans [21] but in animals as well [66]. These findings are in agreement with recent studies which found that dACC cortical Glx plays an important role in modulating the blood-oxygen-level-dependent (BOLD) signal and functional connectivity [5, 6, 67, 68], and extend the literature by demonstrating that Glx/Glutamate may also be relevant for resting state network pathology in psychosis spectrum disorders [11]. Examining group interactions, we noted that higher dACC Glx levels were associated with greater positive FC in the DMN as well as lower positive and negative FC in the ECN in controls, while the opposite was found in FEP, again suggesting that atypical coupling of large-scale networks may be affected by Glx. A recent meta-analysis by Zahid and colleagues where the neurofunctional correlates of glutamate in psychosis were examined revealed six studies using resting state fMRI and dACC cortical Glx and two did not find significant group interactions [10, 69], and the remaining four found group interactions like in our current analysis [13, 25, 70, 71]. More specifically, our previous findings [13] and Limongi’s of weaker FC-Glx interactions are consistent with these findings. From a neuroenergetics perspective, the brain uses large amounts of energy to support synaptic transmission among distal brain regions. This process is highly dependent on a critical balance between glucose (a proxy of brain activity) and glutamatergic activity, which makes this energy demand very efficient [72]. Therefore, these abnormalities observed in FC-Glx interactions may be a result of abnormal glucose and Glx activity in FEP.

Several strengths and limitations of this study are notable. We enrolled a large group of antipsychotic-naïve FEP patients, which allowed us to mitigate antipsychotic medication or illness chronicity confounds. We decided not to exclude patients with a history of cannabis use, as it is a major risk factor for developing psychosis and thus highly clinically relevant, and excluding these patients would have inadvertently biased our sample and limited the generalizability of our data. Our 1H-MRS acquisition sequence does have some drawbacks such as J-modulation and T2 relaxation effects on the spectrum as well as a water signal that is highly T2-weighted and sensitive to cerebrospinal fluid contamination [37], a significant advantage of these acquisition parameters is that it allows us put findings in the context of a number of our previous studies for which we used the same acquisition parameters [73–75]. Moreover, we use tissue correction to reduce this bias. Finally, Glx was quantified instead of glutamate, therefore, our findings may not completely generalize to strictly glutamatergic abnormalities in psychosis, the heterogeneity within our FEP sample, and associations between symptom severity and imaging variables.

## Conclusions

In summary, we extended the literature by demonstrating that both correlations and anticorrelations in large-scale resting-state networks supporting higher-order cognition are already altered in antipsychotic-naïve patients suffering from a first psychotic episode, underscoring the importance of also considering anticorrelations for optimal characterization of higher-order functional brain networks in this population. Our data also adds to the growing body of evidence supporting the role of dACC cortical Glx as a mechanism underlying alterations in functional connectivity. Given the considerable illness burden in psychosis spectrum disorders and the limited efficacy of conventional antipsychotic medications, future studies investigating novel agents attenuating connectivity alterations, possibly via modulation of glutamatergic pathways, are direly needed in the field.

### Supplementary information

Supplementary figures

Supplementary tables

## Data Availability

The dataset here has subject overlap with our previous study [13] and recent reports [76–79]. Data for NCT 034420101 is deposited to the NDA data archive and shared per NIMH agreement.
