# Supplementary material for: Higher-order functional brain networks and anterior cingulate glutamate + glutamine (Glx) in antipsychotic-naïve first episode psychosis patients
Source: Transl Psychiatry. 2024 Apr 10;14:183. doi: 10.1038/s41398-024-02854-7 (PMC11006887; doi:10.1038/s41398-024-02854-7)
Supplement: Supplementary file 1 — Supplementary figures [file 41398_2024_2854_MOESM1_ESM.pdf]

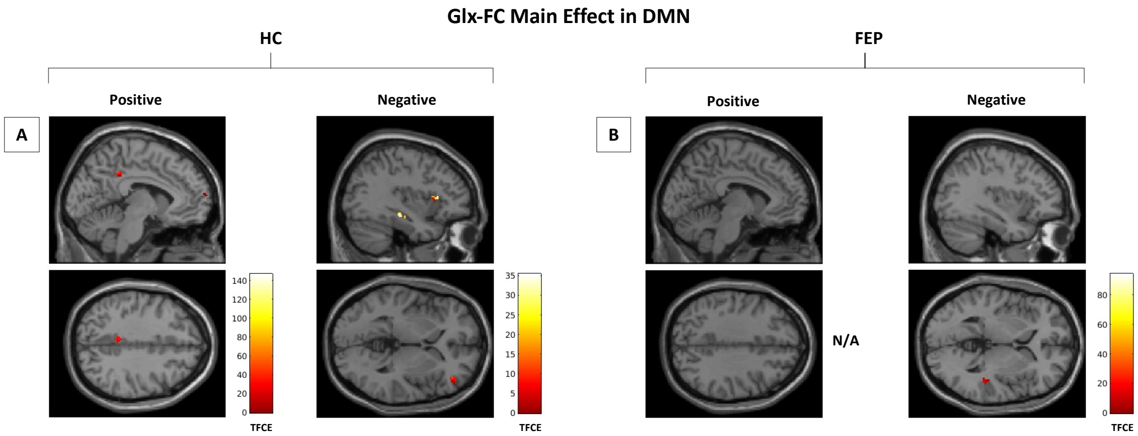

Supplementary figure 1. Clusters indicate correlations between positive/negative FC and Glx in A) HC and B) FEP in the DMN (TFCE corrected). DMN, default mode network; TFCE, threshold-free cluster enhancement; HC, healthy controls; FEP, first episode psychosis.

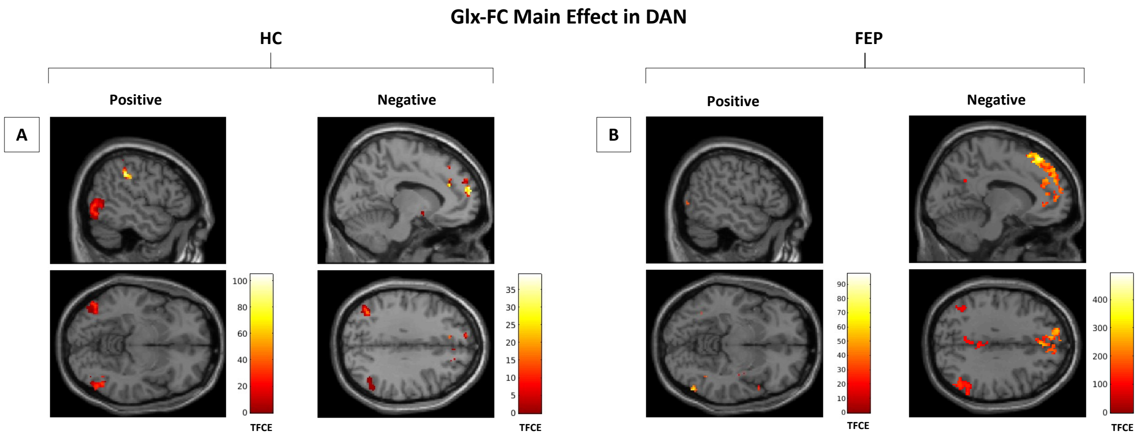

Supplementary figure 2. Clusters indicate correlations between positive/negative FC and Glx in A) HC and B) FEP in the DAN (TFCE corrected). DAN, dorsal attention network; TFCE, threshold-free cluster enhancement; HC, healthy controls; FEP, first episode psychosis.

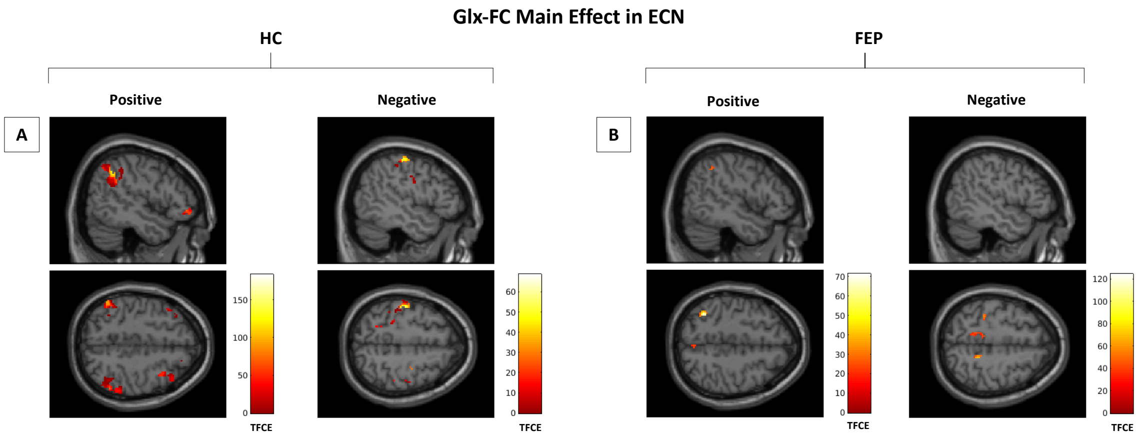

Supplementary figure 3. Clusters indicate correlations between positive/negative FC and Glx in A) HC and B) FEP in the ECN (TFCE corrected). ECN, executive control network; TFCE, threshold-free cluster enhancement; HC, healthy controls; FEP, first episode psychosis.
